# Supplementary material for: OrgaSegment: deep-learning based organoid segmentation to quantify CFTR dependent fluid secretion
Source: Commun Biol. 2024 Mar 13;7:319. doi: 10.1038/s42003-024-05966-4 (PMC10937908; doi:10.1038/s42003-024-05966-4)
Supplement: Supplementary file 2 — Supplementary Information [file 42003_2024_5966_MOESM2_ESM.pdf]

Lefferts et al.,Supplementary Figure 1

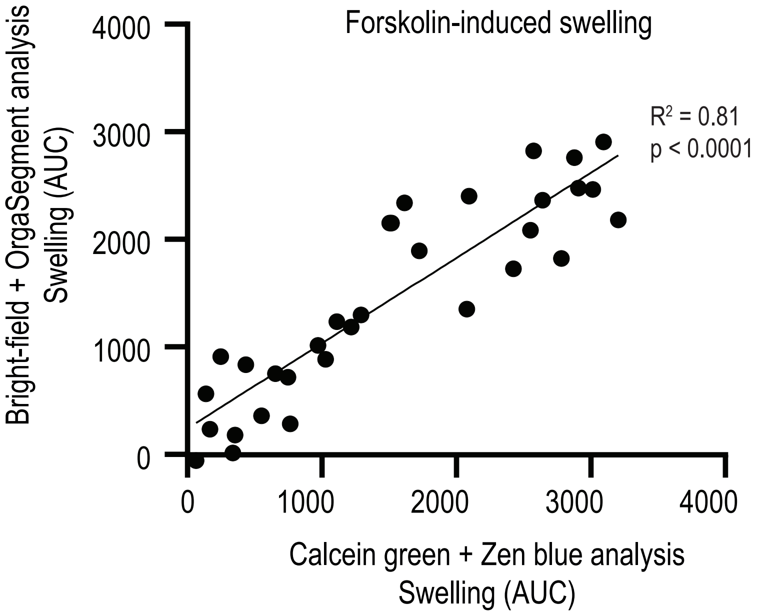

Supplementary figure 1: FIS analysis from bright-field images using OrgaSegment versus conventional calcein green analysis using Zeiss Zen blue software.

FIS calculated from calcein green images obtained with a Zeiss LSM800 microscope and analyzed via standard Zeiss Zen blue analysis pipeline (x-axis) versus FIS calculated from bright-field images obtained with a Leica Thunder microscope, segmented with OrgaSegment (y-axis). FIS experiments are performed on three different donors with various experimental conditions, each dot represents an experimental condition.

Lefferts et al., Supplementary Figure 2

a

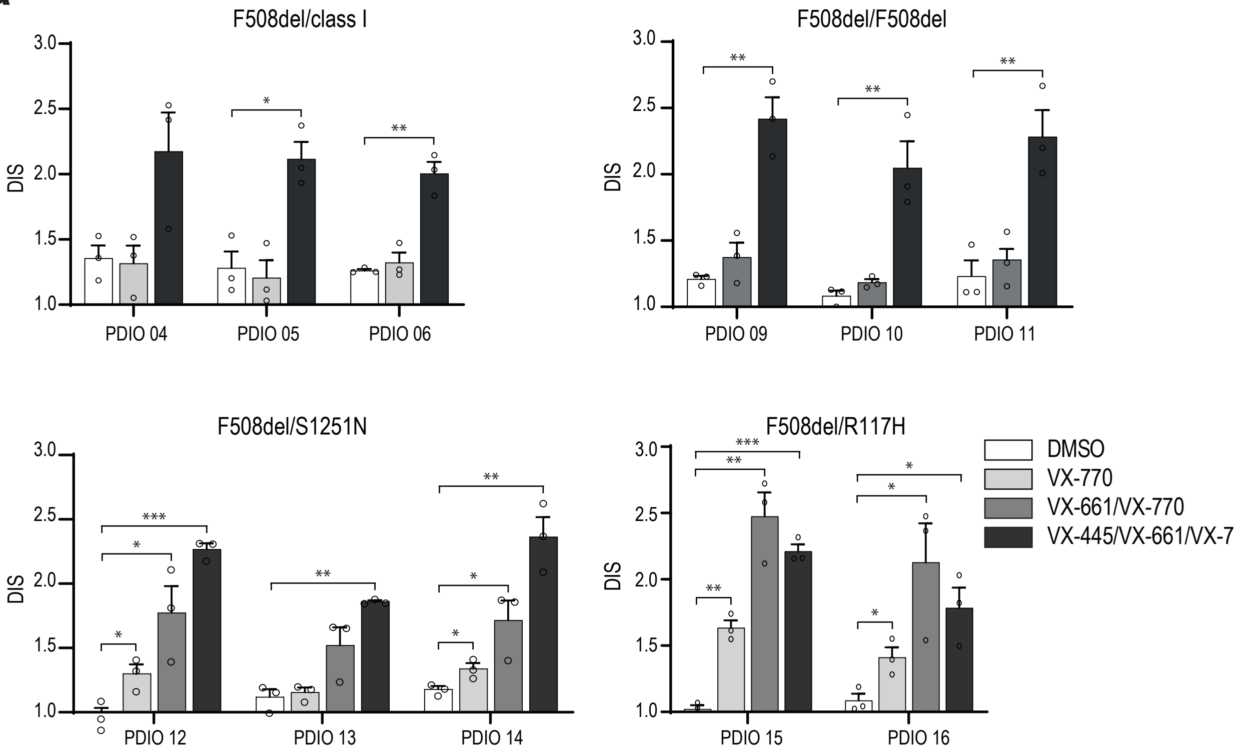

b

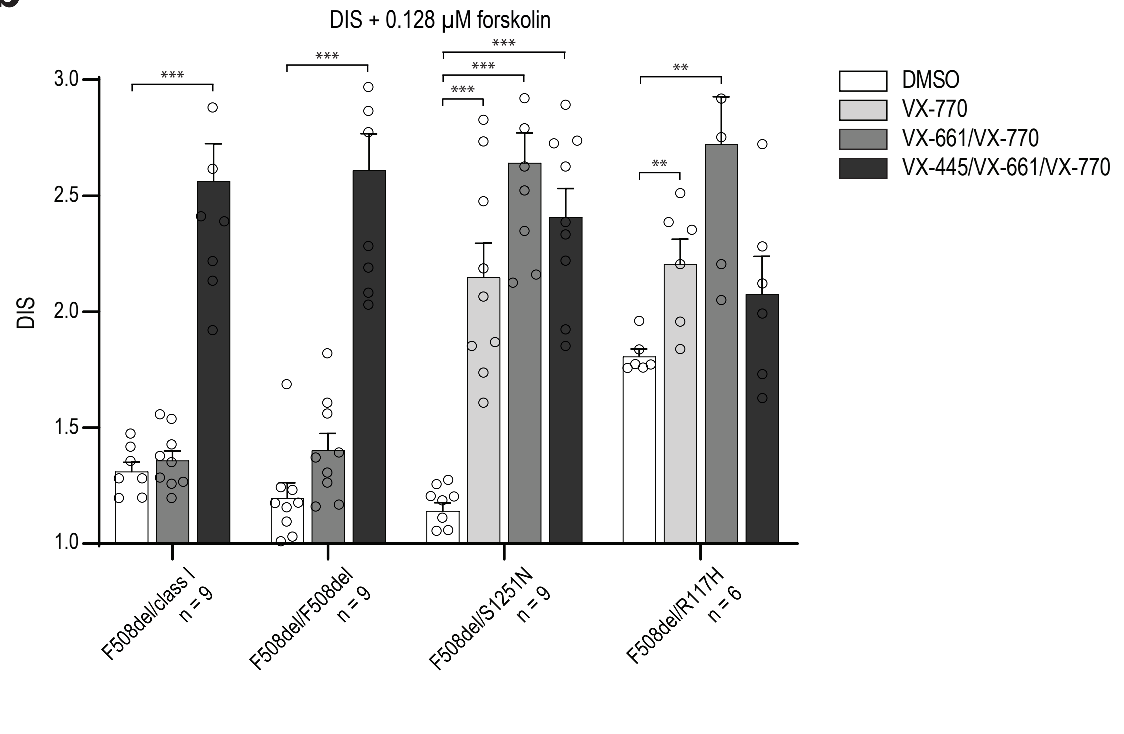

Supplementary figure 2: DIS response of individual donors and DIS response in the presence of forskolin stimulation.

a. DIS response of individual donors for various *CFTR* genotypes. All experiments are performed in biological triplicates. Error bars represent the standard error of the mean and significance, calculated using two-tailed t-tests, is compared to vehicle (DMSO) treated conditions (* $p < 0.05$, ** $p < 0.01$, *** $p < 0.001$).
b. DIS response in the presence of 0.128 µM overnight forskolin treatment. Experimental size n equals the amount of donors times three experimental replicates. Error bars represent the standard error of the mean and significance, calculated using two-tailed t-tests, is compared to vehicle (DMSO) treated conditions (* $p < 0.05$, ** $p < 0.01$, *** $p < 0.001$).

Lefferts et al., Supplementary Figure 3

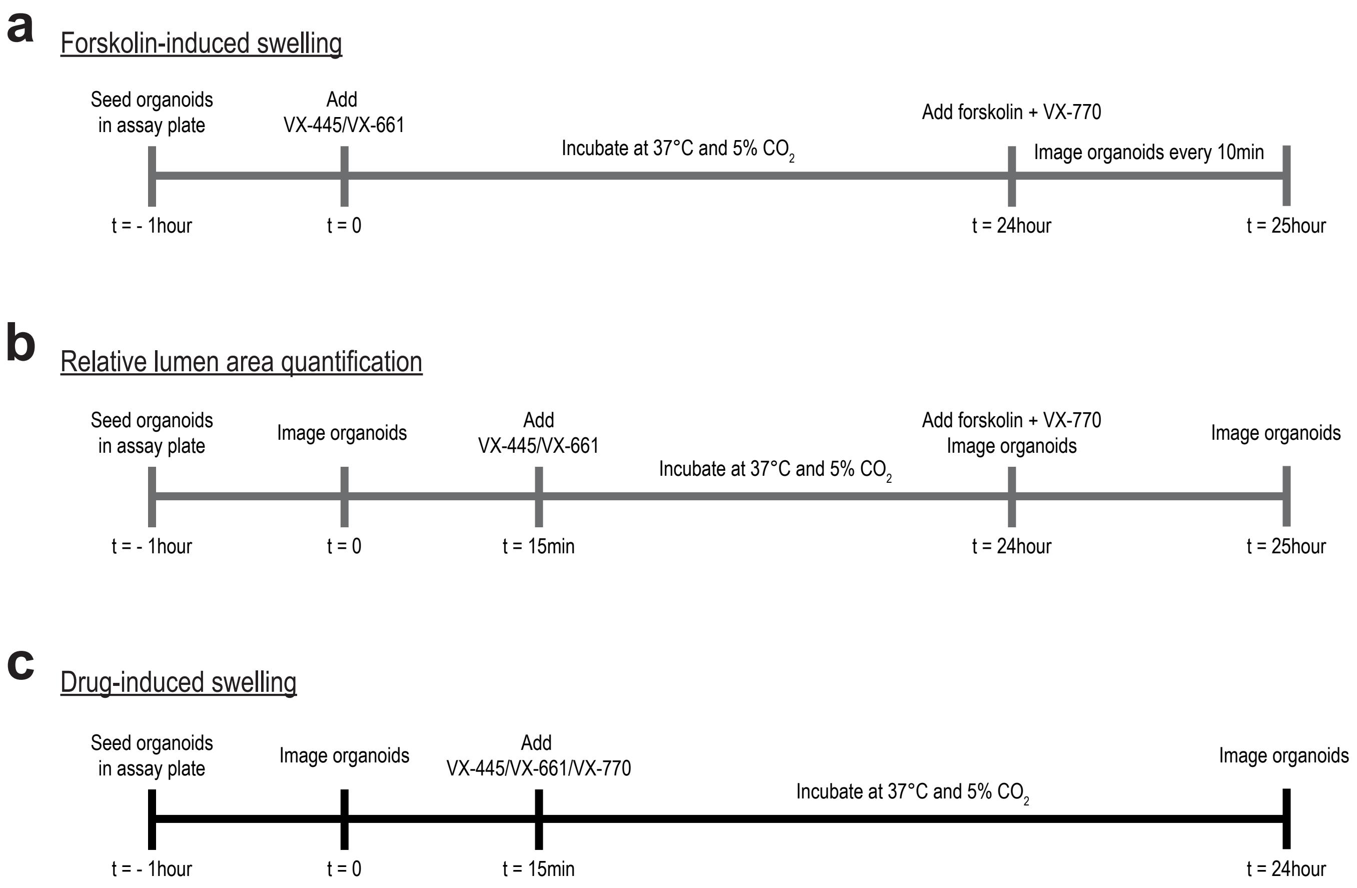

Supplementary figure 3: Schematic overview of the timelines of the various functional CFTR function measurements used in this study.

a. Timeline (depicted in grey) of the forskolin-induced swelling assay following standard protocol[13].
b. Timeline (depicted in grey) of relative lumen area measurements, adapted from the steady-state lumen area protocol[15].
c. Timeline (depicted in black) of the drug-induced swelling assay, as developed in this paper.

Lefferts et al., Supplementary table 1: List of *CFTR* mutations per donor.

Identifier	*CFTR* mutation 1	*CFTR* mutation 2
**PDIO 01**	A46D	A46D
**PDIO 02**	Q1012P	N1303K
**PDIO 03**	R1066H	CFTRdele2,3
**PDIO 04**	F508del	E730X
**PDIO 05**	F508del	Y1092X
**PDIO 06**	F508del	W1282X
**PDIO 07**	F508del	R1162X
**PDIO 08**	F508del	Y1092X
**PDIO 09**	F508del	F508del
**PDIO 10**	F508del	F508del
**PDIO 11**	F508del	F508del
**PDIO 12**	F508del	S1251N
**PDIO 13**	F508del	S1251N
**PDIO 14**	F508del	S1251N
**PDIO 15**	R117H	F508del
**PDIO 16**	R117H	F508del
