# Supplementary material for: OrgaSegment: deep-learning based organoid segmentation to quantify CFTR dependent fluid secretion
Source: Commun Biol. 2024 Mar 13;7:319. doi: 10.1038/s42003-024-05966-4 (PMC10937908; doi:10.1038/s42003-024-05966-4)
Supplement: Supplementary file 3 — Reporting Summary [file 42003_2024_5966_MOESM3_ESM.pdf]

nature portfolio

Corresponding author(s): S.F.B. van Beuningen and J. Beekman

Last updated by author(s): Jan 22, 2024

Reporting Summary

Nature Portfolio wishes to improve the reproducibility of the work that we publish. This form provides structure for consistency and transparency in reporting. For further information on Nature Portfolio policies, see our Editorial Policies and the Editorial Policy Checklist.

Statistics

For all statistical analyses, confirm that the following items are present in the figure legend, table legend, main text, or Methods section.

n/a	Confirmed	
☐	☒	The exact sample size (*n*) for each experimental group/condition, given as a discrete number and unit of measurement
☐	☒	A statement on whether measurements were taken from distinct samples or whether the same sample was measured repeatedly
☐	☒	The statistical test(s) used AND whether they are one- or two-sided<br>*Only common tests should be described solely by name; describe more complex techniques in the Methods section.*
☒	☐	A description of all covariates tested
☒	☐	A description of any assumptions or corrections, such as tests of normality and adjustment for multiple comparisons
☐	☒	A full description of the statistical parameters including central tendency (e.g. means) or other basic estimates (e.g. regression coefficient) AND variation (e.g. standard deviation) or associated estimates of uncertainty (e.g. confidence intervals)
☐	☒	For null hypothesis testing, the test statistic (e.g. *F*, *t*, *r*) with confidence intervals, effect sizes, degrees of freedom and *P* value noted<br>*Give P values as exact values whenever suitable.*
☒	☐	For Bayesian analysis, information on the choice of priors and Markov chain Monte Carlo settings
☒	☐	For hierarchical and complex designs, identification of the appropriate level for tests and full reporting of outcomes
☒	☐	Estimates of effect sizes (e.g. Cohen's *d*, Pearson's *r*), indicating how they were calculated

Our web collection on statistics for biologists contains articles on many of the points above.

Software and code

Policy information about availability of computer code

Data collection	The forked and optimized MASK R-CNN code is available at GitHub (release v2.1.3): https://github.com/Living-Technologies/Mask_RCNN (DOI: https://doi.org/10.5281/zenodo.6866648). The OrgaSegment code including configuration, scripts (e.g. training, evaluation, prediction), model weights (.h5 file), application, and README is available at GitHub: https://github.com/Living-Technologies/OrgaSegment
Data analysis	The forked and optimized MASK R-CNN code is available at GitHub (release v2.1.3): https://github.com/Living-Technologies/Mask_RCNN (DOI: https://doi.org/10.5281/zenodo.6866648). The OrgaSegment code including configuration, scripts (e.g. training, evaluation, prediction), model weights (.h5 file), application, and README is available at GitHub: https://github.com/Living-Technologies/OrgaSegment

For manuscripts utilizing custom algorithms or software that are central to the research but not yet described in published literature, software must be made available to editors and reviewers. We strongly encourage code deposition in a community repository (e.g. GitHub). See the Nature Portfolio guidelines for submitting code & software for further information.

Data

Policy information about availability of data

All manuscripts must include a data availability statement. This statement should provide the following information, where applicable:

- Accession codes, unique identifiers, or web links for publicly available datasets
- A description of any restrictions on data availability
- For clinical datasets or third party data, please ensure that the statement adheres to our policy

The training and experimental data that support the findings of this study are available upon request during the manuscript review process and will be made publicly available in Zenodo (https://zenodo.org/communities/livingtech/) upon publication.

Research involving human participants, their data, or biological material

Policy information about studies with human participants or human data. See also policy information about sex, gender (identity/presentation), and sexual orientation and race, ethnicity and racism.

Reporting on sex and gender	n.a.
Reporting on race, ethnicity, or other socially relevant groupings	n.a.
Population characteristics	n.a.
Recruitment	Organoids used were selected based on genotype obtained from the HUB (Hubrecht Organoid Technology) Biobank (www.huborganoids.nl) under TC-Bio protocol number 14-008, or from the UMCU Darmbank under TC-Bio protocol number 19-831, and used according to informed consent.
Ethics oversight	Organoids were used under TC-bio (UMC Utrecht biobanks review committee) approval number 14-008 and 19-831

Note that full information on the approval of the study protocol must also be provided in the manuscript.

Field-specific reporting

Please select the one below that is the best fit for your research. If you are not sure, read the appropriate sections before making your selection.

☒ Life sciences ☐ Behavioural & social sciences ☐ Ecological, evolutionary & environmental sciences

For a reference copy of the document with all sections, see nature.com/documents/nr-reporting-summary-flat.pdf

Life sciences study design

All studies must disclose on these points even when the disclosure is negative.

Sample size	Where possible 3 different donors were studied per genotype. For rare genotypes, only 1 donor was available.
Data exclusions	DIS measurements were less than 32 organoid structures were present excluded. This cut-off was established based on results (figure 3d)
Replication	All measurements were performed in biological triplicate (performed at an independent time-point from a different culture passage)
Randomization	Samples were allocated into experimental group based on genotype.
Blinding	Investigators were not blinded, data analysis was performed in an automated manner

Reporting for specific materials, systems and methods

We require information from authors about some types of materials, experimental systems and methods used in many studies. Here, indicate whether each material, system or method listed is relevant to your study. If you are not sure if a list item applies to your research, read the appropriate section before selecting a response.

Materials & experimental systems

n/a	Involved in the study	
☒	☐	Antibodies
☒	☐	Eukaryotic cell lines
☒	☐	Palaeontology and archaeology
☒	☐	Animals and other organisms
☒	☐	Clinical data
☒	☐	Dual use research of concern
☒	☐	Plants

Methods

n/a	Involved in the study	
☒	☐	ChIP-seq
☒	☐	Flow cytometry
☒	☐	MRI-based neuroimaging
